# Differential Reponses of Hematopoietic Stem and Progenitor Cells to mTOR Inhibition

**DOI:** 10.1155/2015/561404

**Published:** 2015-06-28

**Authors:** Aimin Yang, Xia Xiao, Mingfeng Zhao, Amanda C. LaRue, Bradley A. Schulte, Gavin Y. Wang

**Affiliations:** ^1^Department of Pathology and Laboratory Medicine, Medical University of South Carolina, Charleston, SC 29425, USA; ^2^Department of Hematology, Tianjin First Center Hospital, Tianjin 300192, China; ^3^Research Services, Ralph H. Johnson VAMC, Charleston, SC, USA; ^4^Cancer Genes and Molecular Regulation Program, Hollings Cancer Center, Medical University of South Carolina, Charleston, SC 29425, USA

## Abstract

Abnormal activation of the mammalian target of rapamycin (mTOR) signaling pathway has been observed in a variety of human cancers. Therefore, targeting of the mTOR pathway is an attractive strategy for cancer treatment and several mTOR inhibitors, including AZD8055 (AZD), a novel dual mTORC1/2 inhibitor, are currently in clinical trials. Although bone marrow (BM) suppression is one of the primary side effects of anticancer drugs, it is not known if pharmacological inhibition of dual mTORC1/2 affects BM hematopoietic stem and progenitor cells (HSPCs) function and plasticity. Here we report that dual inhibition of mTORC1/2 by AZD or its analogue (KU-63794) depletes mouse BM Lin^−^Sca-1^+^c-Kit^+^ cells in cultures via the induction of apoptotic cell death. Subsequent colony-forming unit (CFU) assays revealed that inhibition of mTORC1/2 suppresses the clonogenic function of hematopoietic progenitor cells (HPCs) in a dose-dependent manner. Surprisingly, we found that dual inhibition of mTORC1/2 markedly inhibits the growth of day-14 cobblestone area-forming cells (CAFCs) but enhances the generation of day-35 CAFCs. Given the fact that day-14 and day-35 CAFCs are functional surrogates of HPCs and hematopoietic stem cells (HSCs), respectively, these results suggest that dual inhibition of mTORC1/2 may have distinct effects on HPCs versus HSCs.

## 1. Introduction

Mechanistic/mammalian target of rapamycin (mTOR) is a highly conserved serine/threonine protein kinase that belongs to the phosphatidylinositol-3 kinase (PI3K) family and serves as a central regulator of cell metabolism, growth, proliferation, and survival [1, 2]. The mTOR kinase exists in two functionally different complexes, mTOR complex 1 (mTORC1) and mTOR complex 2 (mTORC2) that have distinct substrate molecules involved in the regulation of protein translation and cellular metabolism [3]. It has been shown that mTORC1 stimulates protein translation by phosphorylating downstream targets including 4E-BP1 and p70 ribosomal protein S6 kinase (p70S6K) [4]. In contrast, the functional role of mTORC2 is less clear and it was reported that mTORC2 phosphorylates AGC kinase family members including AKT, SGK1, and PKC*α* [3–5]. Interestingly, aberrant activation of the PI3K/AKT/mTOR signaling pathway has been observed in many types of solid tumors as well as leukemia [6–12]. For example, the PI3K-AKT signaling pathway is frequently activated in patients with T-cell acute lymphoblastic leukemia (T-ALL) as a result of loss-of-function mutation of the phosphatase PTEN, a suppressor of PI3K. Consequently, AKT activates downstream mTORC1 via PRAS40 and the tuberous sclerosis 1/2- (TSC1/2-) Rheb pathway. These observations strongly suggest that targeted inhibition of overactivated mTOR pathway may represent a new and effective strategy for cancer treatment.

Although mTOR was originally identified as a target protein of rapamycin, a natural macrolide immunosuppressant, rapamycin primarily inhibits the kinase activity of mTORC1 and is much less effective in curbing mTORC2 activity [3]. Furthermore, it has been shown that 4E-BP1 is a rapamycin-insensitive mTORC1 substrate, indicating that rapamycin treatment does not necessarily represent a successful blockade of mTORC1 function [16]. Inhibition of mTORC1 by rapamycin and its analogs has been explored to treat various types of human cancers. However, the efficacy of such treatment is limited and it appears that many patients display only modest or even no response to the therapy [17–19]. Therefore, great efforts have been made to identify novel mTOR inhibitors that suppress both mTORC1 and mTORC2 activity. Recently several ATP-competitive inhibitors of mTOR kinase, including INK128 and AZD8055, have been developed and are being evaluated in clinical trials [20–23]. These dual mTORC1/2 inhibitors not only represent potential novel and more effective anticancer therapeutics but also provide valuable research tools for understanding the biology of mTOR.

Given the fact that BM suppression is a significant safety concern for many anticancer drugs, it is important to determine if dual mTORC1/2 inhibition has any adverse effects on BM HSPCs. In this report, we provide data showing that treatment with AZD depletes HSPCs via apoptosis induction. Furthermore, we found that AZD treatment inhibits day-14 CAFCs but promotes day-35 CAFCs, indicating that HSCs and HPCs may have differential responses to mTOR inhibition. Together, these results demonstrate a critical role for mTOR in HSPC survival and suggest that potential BM suppression should be a viable concern for patients who are considering of taking dual mTORC1/2 inhibitors either alone or in combination with other chemotherapeutic agents in the course of cancer treatment.

## 2. Materials and Methods

### 2.1. Reagents

KU-63794 was obtained from CalBiochem and AZD8055 was purchased from Selleckchem. Phycoerythrin (PE) Cy7-conjugated anti-Sca-1 (Clone E13-161.7, rat IgG2a), APC-conjugated anti-c-kit (Clone 2B8, rat IgG2b), and purified rat anti-CD 16/CD32 (Clone 2.4G2, Fc*γ* receptor blocker, rat IgG2b) were purchased from BD Pharmingen (San Diego, CA). Both mouse and human Hematopoietic Progenitor (Stem) Cell Enrichment Set-DM were purchased from BD Biosciences. Recombinant mouse thrombopoietin (TPO) was purchased from R&D Systems (Minneapolis, MN). The rabbit anti-phospho-p70 S6 kinase (p70S6K) monoclonal antibody and active (cleaved) caspase-3 antibodies were purchased from Cell Signaling. The Alexa Fluor 594-conjugated goat anti-rabbit IgG antibody was purchased from Invitrogen (Carlsbad, CA).

### 2.2. Mice

Male C57BL/6 mice were purchased from The Jackson Laboratories (Bar Harbor, ME). Mice were housed four to five per cage at the Medical University of South Carolina (MUSC) AAALAC-certified animal facility. They received food and water ad libitum. All mice were used at approximately 8 to 12 weeks of age. The Institutional Animal Care and Use Committee of MUSC approved all experimental procedures used in this study.

### 2.3. Isolation of BM Mononuclear Cells (BM-MNCs) and Lineage Negative HSPCs

BM-MNCs were isolated as we described previously [14, 15]. Lineage negative HSPCs (Lin^−^ HSPCs) were enriched and purified using a mouse Progenitor and Stem Cell Enrichment Kit (BD Biosciences) according to the manufacturer's protocol [13]. Briefly, mouse BM-MNCs were labeled with the biotinylated mouse lineage depletion cocktail containing monoclonal antibodies against mouse CD3e, CD11b, CD45R/B220, Gr-1, and Ter-119. Cells committed to the T- and B-lymphocytic, myeloid, and erythroid lineages were then depleted by MACS using the BD IMag streptavidin magnetic beads (BD Biosciences).

### 2.4. Immunofluorescent Microscopic Analysis of p70S6K Phosphorylation

Activation of mTOR was evaluated by examination of the phosphorylation of mTORC1 substrate, p70S6K, in HSPCs using immunofluorescence microscopic analysis. Briefly, Lin^−^ HSPCs were cultured in a 12-well plate and treated with TPO (100 ng/mL) in the presence or absence of AZD (100 nM). At 60 min after TPO treatment, cells were harvested from culture and cytospun onto slides. The cells were then fixed with 4% paraformaldehyde and permeabilized with 0.2% Triton X-100. Slides were incubated with rabbit anti-phosphorylated p70S6K (pS6K) monoclonal antibody overnight at 4 degrees. The pS6K labeling was visualized using an Alexa Fluor 594-conjugated goat anti-rabbit IgG antibody. Nuclei were counterstained with DAPI. The images were captured and processed using a Zeiss Axio Observer Z1 microscope.

### 2.5. Flow Cytometric Apoptosis Assay

Lin^−^ cells were cultured in IMDM medium containing 10% FBS and 20 ng/mL mouse recombinant TPO and treated with different dose of AZD and KU-63974 or DMSO as a vehicle control. Approximately 20 h after treatment, cells were collected and stained with PE-Cy7-conjugated anti-Sca1 and APC-conjugated anti-c-kit antibodies. Cells were then stained with FITC-Annexin V and 7AAD using an apoptosis assay kit (BD Biosciences) according to the manufacturer's protocol. Apoptotic cells were determined by flow cytometric analysis on an LSRFortessa (Becton Dickinson, San Jose, CA) and the data were analyzed using FlowJo software.

### 2.6. Colony-Forming Unit (CFU) and Cobblestone Area-Forming Cell (CAFC) Assays

CFU assays were performed by culturing BM-MNCs in MethoCult GF M3434 methylcellulose medium (Stem Cell Technologies, Vancouver, BC) according to the manufacturer's protocol. Colonies of colony-forming unit-granulocyte macrophage (CFU-GM) and burst-forming unit-erythroid (BFU-E) were scored on day-7, while colonies of CFU-granulocyte, -erythrocyte, -monocyte, and -megakaryocyte (CFU-GEMM) were enumerated on day-12 after incubation. CAFC assays were conducted to evaluate HSC activity* in vitro* as we reported previously [14]. And day-14 and day-35 CAFC frequencies were determined to measure the functions of HPCs and HSCs, respectively, as described previously [13–15].

### 2.7. Statistical Analysis

Comparisons between different groups were carried out using Student's *t*-test. Differences were considered statistically significant at *p* < 0.05. All analyses were carried out with the GraphPad Prism program (GraphPad Software, Inc., San Diego, CA).

## 3. Results

### 3.1. AZD Treatment Inhibits TPO-Induced Activation of the mTOR Signaling Pathway in HSPCs

**Figure 1:**
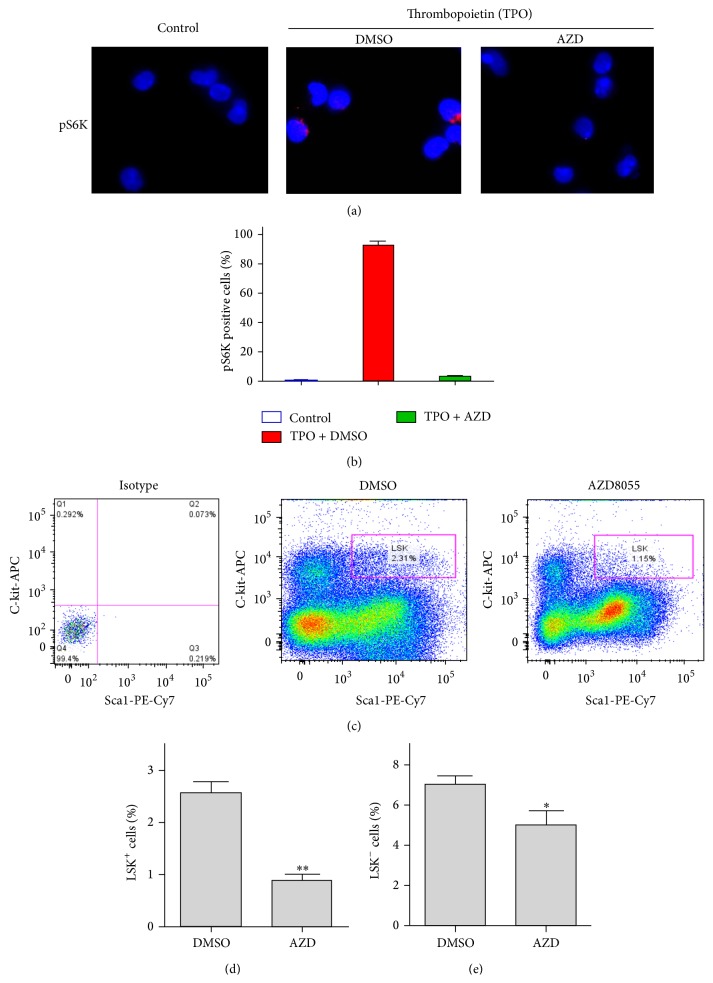
Inhibition of mTOR depletes HSPCs in culture. (a) Phosphorylated S6K (pS6K) was determined using a pS6K specific monoclonal antibody and immunofluorescence microscopy. (b) The percentage of pS6K positive cells was calculated and is presented as mean +/− SEM. (c) The frequency of Lin^−^Sca-1^+^c-Kit^+^ (LSK^+^) cells (HSCs/MPPs enriched subpopulations) and Lin^−^Sca-1^−^c-Kit^+^ (LSK^−^) cells (HPCs) in cultures after AZD treatment was determined by flow cytometric analyses and representative flow cytometric graphs are presented. (d) The frequency of LSK^+^ cells in cultured mouse BM Lin^−^ cells at 20 h after AZD treatment is presented as mean +/− SEM. (e) The frequency of HPCs in in cultured mouse BM Lin^−^ cells at 20 h after AZD treatment is presented. Data are presented as mean +/− SEM of three independent experiments.^*∗*^ *p* < 0.05; ^*∗∗*^ *p* < 0.01 versus DMSO vehicle control.

Previous studies have shown that AZD is a potent inhibitor of mTOR that inhibits p70S6 kinase (S6K) phosphorylation [24]. Evidence that TPO treatment activates S6K and the mTOR signaling pathway in hematopoietic cells also exists [25, 26]. However, it remains to be determined if AZD treatment affects the activity of mTOR signaling in HSPCs. Here, we show that TPO treatment markedly induces the activation of S6K in HSPCs as demonstrated by increased expression of phosphorylated S6K following TPO treatment (Figures 1(a) and 1(b)). More importantly, immunofluorescent studies revealed that AZD treatment almost completely inhibits TPO-induced phosphorylation of S6K (Figures 1(a) and 1(b)). Given that S6K is a key downstream target of mTOR signaling, these findings demonstrate that AZD inhibits TPO-induced activation of the mTOR pathway in HSPCs.

### 3.2. Inhibition of mTOR Depletes HSPCs in Culture by Inducing Apoptosis

It has been recently shown that mTORC1 is required for HSC self-renewal function [26]. However, it is largely unknown if and to what extent pharmacological inhibition of mTOR affects HSPCs. To address this important issue, we employed AZD as a potent mTORC1/2 dual inhibitor to investigate the effects of mTOR inhibition on HSPC survival. The results reveal that AZD treatment significantly reduces the frequency of Lin^−^Sca-1^+^c-Kit^+^ (LSK^+^) and Lin^−^Sca-1^−^c-Kit^+^ (LSK^−^) cells (Figures 1(c)–1(e)). As LSK^+^ cells are enriched for HSCs and multipotent progenitors (MPPs), these data demonstrate that mTOR is required for HSCs/MPPs survival and that dual inhibition of both mTORC1 and mTORC2 leads to the depletion of HSPCs in culture.

**Figure 2:**
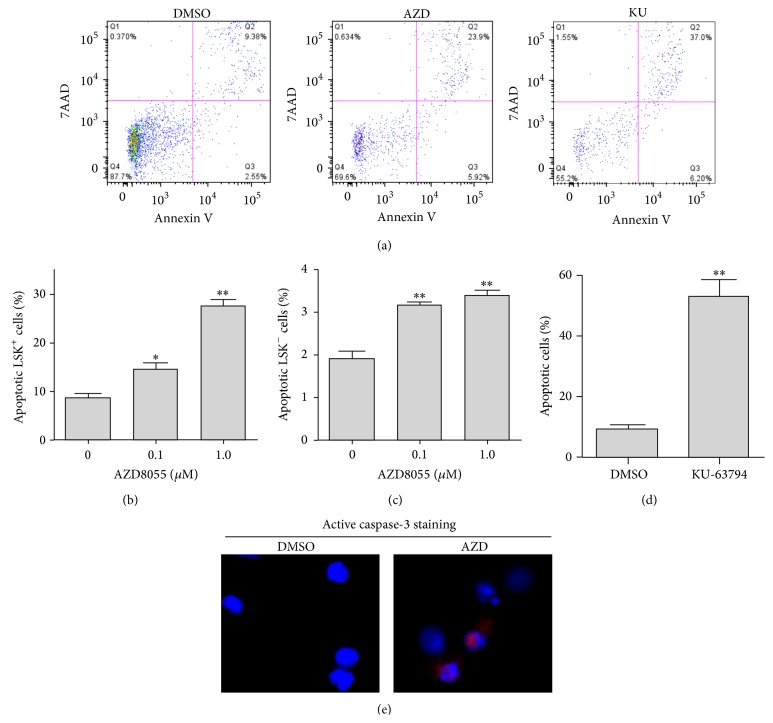
Inhibition of mTOR induces apoptosis in HSPCs. (a) Representative flow cytometric graphs of apoptosis assays using an apoptosis analysis kit as we previously reported [13]. (b) The percentage of apoptotic cells in LSK^+^ subpopulations after different doses of AZD treatment is presented. (c) The percentage of apoptotic cells in LSK^−^ subpopulations after different doses of AZD treatment is presented. (d) The percentage of apoptotic LSK^+^ cells in cultured mouse BM Lin^−^ cells after KU-63794 (10 *μ*M) treatment is presented. (e) Mouse lineage negative HSPCs were treated with AZD (0.1 *μ*M) or DMSO as vehicle control for 16 h. Active caspase-3 immunostaining was performed to determine apoptotic cells in HSPCs. Representative photomicrographs of active caspase-3 immunofluorescent staining (red) and nucleic counterstaining with DAPI (blue) are shown. Data are presented as mean +/− SEM of three independent experiments. ^*∗*^ *p* < 0.05; ^*∗∗*^ *p* < 0.01 versus DMSO vehicle control.

To further gain insight into the mechanisms whereby mTOR inhibition depletes HSPCs, we investigated if AZD treatment causes cell death in HSPCs. Flow cytometric analyses reveal that the percentage of apoptotic cells is markedly increased in LSK^+^ cells after AZD treatment, compared with cells treated with DMSO as vehicle control (Figures 2(a) and 2(b) and Supplementary Figure S1, in Supplementary Material available online at http://dx.doi.org/10.1155/2015/561404). Interestingly, we also found that the basal level of apoptosis in LSK^−^ cells (HPCs) is much lower than that in LSK^+^ cells (Figures 2(b) and 2(c)). Moreover, the data also indicate that AZD induces apoptosis in HPCs in a dose-dependent manner (Figure 2(c)). Similar results were observed in KU-63794 treated mouse BM Lin^−^ cells (Figure 2(d)). To verify the ability of AZD to induce apoptosis in HSPCs, we performed immunostaining assays for activated caspase-3. The results showed that AZD treatment significantly increases the number of active caspase-3 positively stained HSPCs compared with cells treated with DMSO as a vehicle control (Figure 2(e)). Together, these results suggest that dual inhibition of mTORC1/2 depletes HSPCs by the induction of apoptotic cell death.

### 3.3. Treatment with Dual mTORC1/2 Inhibitor Suppresses the Clonogenic Function of HPCs

**Figure 3:**
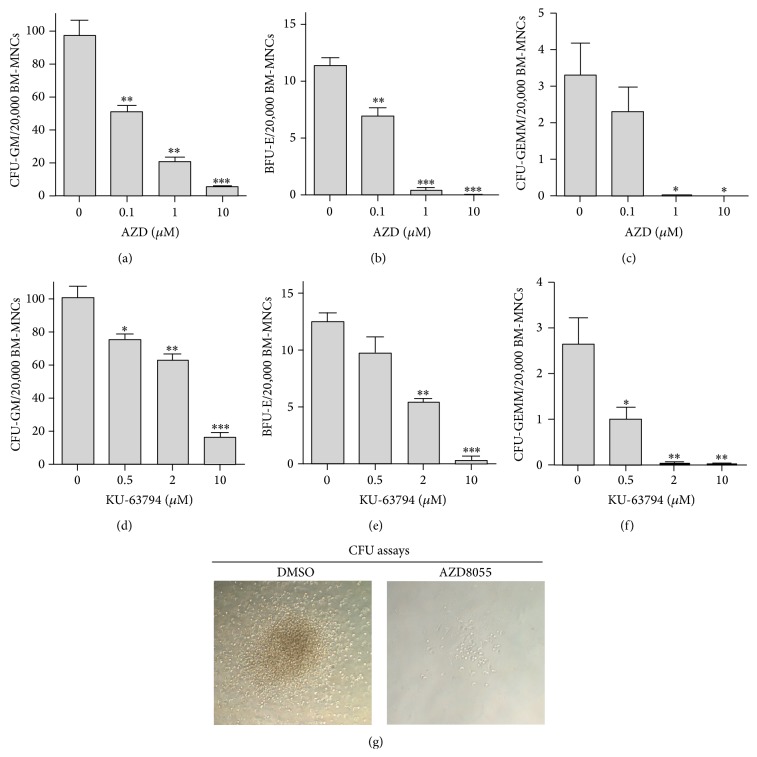
Treatment with dual mTORC1/2 inhibitors suppresses the clonogenic function of HPCs. Colony-forming unit (CFU) assays were performed to determine the functions of HPCs to generate CFU-E, BFU-E, and CFU-GEMM as we previously reported [14, 15]. (a–c) The effects of AZD on HPC functions were determined by CFU assays. (d–f) The effects of KU-63794 on HPC functions were determined by CFU assays. (g) Representative photomicrographs of CFU assays are shown. Data are presented as mean +/− SEM of three independent experiments. ^*∗*^ *p* < 0.05; ^*∗∗*^ *p* < 0.01; ^*∗∗∗*^ *p* < 0.001 versus DMSO vehicle control.

Next, we asked if dual mTORC1/2 inhibition has any impacts on HPC function. To address this question, CFU assays were performed to determine the colony-forming activity of HPCs. As shown in Figures 3(a)–3(c), treatment with AZD resulted in a dose-dependent decrease in the number of CFU-GMs, BFU-Es, and CFU-GEMMs, compared with those treated with DMSO as a vehicle control. Similar results were observed in cells treated with an AZD analogue, KU-63794 (Figures 3(d)–3(f)). It is worth noting that the size of CFU-GM, BFU-E, and CFU-GEMM generated from cells treated with AZD was much smaller than those produced by the control cells treated with DMSO (Figure 3(g) and data not shown). Moreover, our recent data indicated that AZD treatment significantly inhibits the clonogenic function of human cord blood-derived HPCs in a dose-dependent manner (Supplementary Figure S2). These new findings demonstrate for the first time that pharmacological inhibition of both mTORC1 and mTORC2 by small-molecule inhibitors suppresses the colony-forming ability of HPCs.

### 3.4. Dual Inhibition of mTORC1/2 Suppresses Day-14 CAFCs but Promotes Day-35 CAFCs

**Figure 4:**
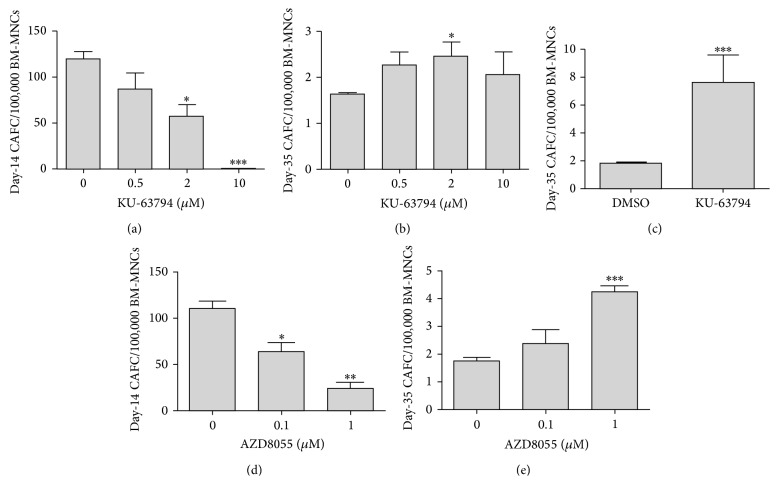
Dual inhibition of mTORC1/2 suppresses day-14 CAFCs but promotes the production of day-35 CAFCs. The functions of HSPCs were determined* in vitro* using CAFC assays as we previously reported [13–15]. (a) Day-14 CAFCs were analyzed to assess the function of HPCs. (b) Day-35 CAFCs were measured to evaluate the function of HSCs* in vitro*. (c) Day-35 CAFCs were determined using the same protocol as shown in (b), except that KU-63974 was readded to the culture medium weekly for 4 weeks. (d) Day-14 CAFCs were performed to assess the function of HPCs in the presence of different doses of AZD. (e) Day-35 CAFCs were measured to evaluate the function of HSCs with or without AZD treatment. Data are presented as mean +/− SEM of three independent experiments. ^*∗*^ *p* < 0.05 versus DMSO vehicle control; ^*∗∗*^ *p* < 0.01; ^*∗∗∗*^ *p* < 0.001 versus DMSO vehicle control.

HSPCs can continuously produce CAFCs in long-term BM culture* in vitro* and the day-35 CAFC assay is a well-established technology to measure HSC functionality* in vitro* [13–15]. To determine if dual mTORC1/2 inhibition affects HSC function, we investigated the effects of KU-63794 treatment on the generation of CAFCs. The results show that KU-63794 treatment leads to a dose-dependent decline in day-14 CAFCs, suggesting that inhibition of mTOR suppresses the function of HPCs in a dose-dependent manner (Figure 4(a)). Interestingly, the number of day-35 CAFCs was not changed to the same extent as that of day-14 CAFCs (Figure 4(b)), indicating that the suppressive effect of KU-63794 on long-term HSCs is likely very limited. More importantly, we found that repeated treatment with low dose of KU-63794 (2 *μ*M, added weekly to culture for 4 weeks) actually enhances the generation of day-35 CAFCs (Figure 4(c)). In addition, our data also show that AZD treatment inhibits day-14 CAFCs but enhances the production of day-35 CAFCs (Figures 4(d) and 4(e)). These new findings demonstrate for the first time that dual mTORC1/2 inhibition displays differential effects on day-14 and day-35 CAFCs, suggesting that HSCs and HPCs may have distinct responses to mTOR inhibition.

## 4. Discussion

HSCs primarily reside in bone morrow and are responsible for the continuous regeneration of all types of blood cells to replenish old and damaged blood cells. Previous studies have demonstrated a critical role for mTOR signaling in HSC function and hematopoiesis [10, 25–27]. For example, it has been recently shown that mTORC1 function is required for HSC regeneration and that loss of mTOR function leads to BM failure and pancytopenia [27, 28]. These observations strongly support the hypothesis that the blockade of mTOR function by ATP-competitive inhibitors such as KU-63794 and AZD may have adverse effects on HSPCs. In this study, we show that inhibition of mTOR by AZD depletes LSK cells by the induction of apoptosis, suggesting that mTOR function is required to maintain HSPC survival in culture. In agreement with this finding, it was reported that genetic silencing of mTOR causes apoptosis in hematopoietic cells, resulting in impaired HSC self-renewal and BM failure [28]. Together, these studies demonstrate that mTOR signaling plays a critical role in regulating the activity and function of HSPCs, suggesting that hematopoietic toxicity would be a concern for the application of mTOR inhibitors in cancer treatment.

In addition, the present study demonstrates for the first time that mTOR inhibition by KU-63794 or AZD preferentially suppresses day-14 CAFCs, but not day-35 CAFCs. These results indicate that dual inhibition of both mTOC1 and mTORC2 may selectively suppress the function of HPCs while sparing HSCs. More interestingly, we found that a low dose of KU-63794 treatment promotes the generation of day-35 CAFCs in long-term BM culture. These findings imply that a modest inhibition of mTORC1/2 functions may benefit the self-renewal capacity of HSCs. Consistent with the above findings, it has been shown that inhibition of mTORC1 function by rapamycin attenuates PTEN loss-induced exhaustion of HSCs and promotes the* ex vivo* expansion of HSCs [7, 29]. Moreover, it was reported that inhibition of mTOR and GSK3*β* activates canonical Wnt-*β*-catenin signaling and enhances the maintenance of long-term HSCs during* ex vivo* culture [30]. Nevertheless, further studies are needed to better understand the mechanisms underlying HSC and HPC's differential responses to the treatment of mTORC1/2 dual inhibitors.

In order to further understand how human HPSCs respond to mTORC1/2 inhibition, we investigated the effects of AZD8055 treatment on the colony-forming activity of human cord blood-derived HPCs. CFU assays revealed that AZD8055 inhibits the clonogenic function of human HPCs in a dose-dependent fashion. Interestingly, it was reported that CD34^+^ human hematopoietic cells appear to be resistant to AZD8055-induced toxicity [31]. Since CD34^+^ cells are enriched for human HSCs, together these data indicate that, like in mouse HSPCs, dual mTORC1/2 inhibition may also have differential effects on human HSCs versus HPCs. From a clinical perspective, these studies suggest that the administration of mTORC1/2 dual inhibitors may cause BM suppression in some patients due to the induction of apoptosis in HPCs. However, this undesired BM suppressive side effect is likely only transient and would be relieved after treatment cessation or following a dose reduction because long-term HSCs may be more resistant to these inhibitors and thus would be able to regenerate new HPSCs and their progenies to replenish those lost hematopoietic cells caused by mTOR inhibition.

## Supplementary Material

The supplementary materials provide a schematic illustration of gating strategy for apoptosis analyses in different subpopulations of HSPCs, and data demonstrating that pharmacological inhibition of mTORC1/2 by AZD8055 suppresses the colony-forming activity of human cord blood-derived HSPCs.
Fig. S1. Schematic illustration of apoptosis analysis of mouse BM HSPCs. Shown are gating strategies for analyzing apoptosis in different subpopulations of HSPCs using an apoptosis detection kit (BD Biosciences) along with flow cytometric analyses.
Fig. S2. AZD8055 inhibits the clonogenic function of human cord blood-derived HPCs. Human cord blood lineage negative (Lin- CB) cells were isolated using a human HSPC enrichment kit (BD Biosciences) by depleting cells expressing myeloid-, erythroid-, and T- and B-lineage makers according to the manufacturer's instructions. CFU assays were performed to assess the number and functions of human HPCs. (A) The effects of AZD on CFU-GM colony production are shown. (B) The effects of AZD on BFU-E colony production are shown. (C) The effects of AZD on CFU-GEMM colony production are shown.∗*p* < 0.05; ∗∗*p* < 0.01; ∗∗∗*p* < 0.001 vs. DMSO vehicle control.
