# Supplementary figures and images for: Differential Reponses of Hematopoietic Stem and Progenitor Cells to mTOR Inhibition
Source: Stem Cells Int. 2015 Jun 28;2015:561404. doi: 10.1155/2015/561404 (PMC4499403; doi:10.1155/2015/561404)

Fig. S1

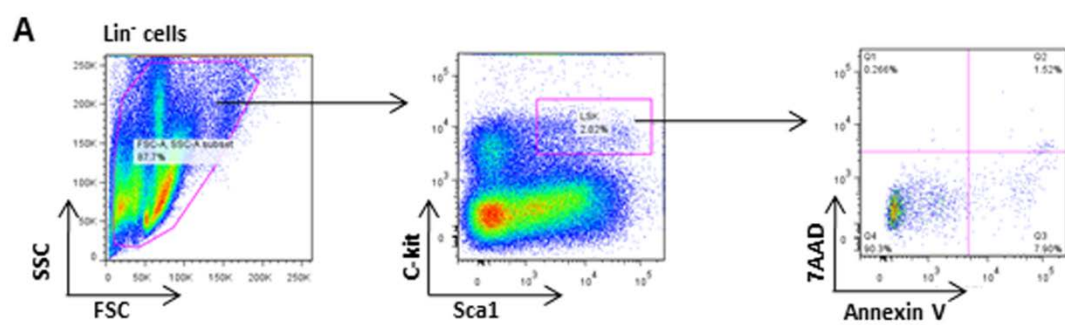

Fig. S2

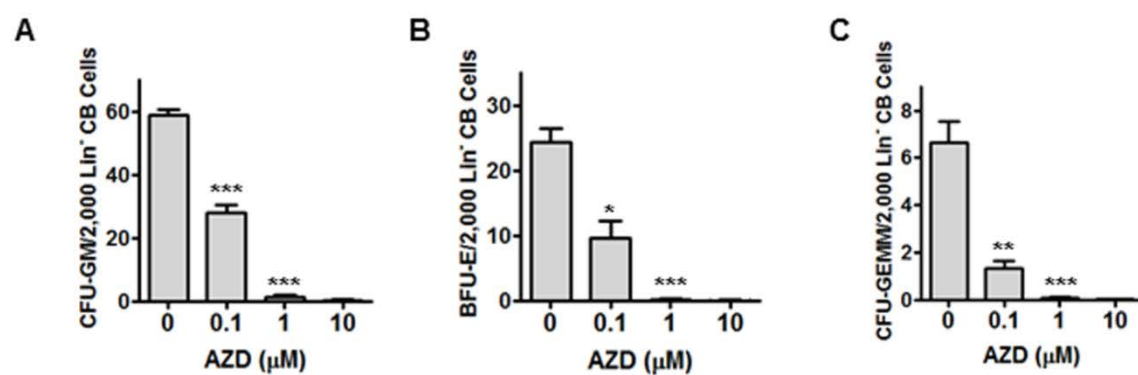

Supplement: Supplementary file 1 — The supplementary materials provide a schematic illustration of gating strategy for apoptosis analyses in different subpopulations of HSPCs, and data demonstrating that pharmacological inhibition of mTORC1/2 by AZD8055 suppresses the colony-forming activity of human cord blood-derived HSPCs. Fig. S1. Schematic illustration of apoptosis analysis of mouse BM HSPCs. Shown are gating strategies for analyzing apoptosis in different subpopulations of HSPCs using an apoptosis detection kit (BD Biosciences) along with flow cytometric analyses. Fig. S2. AZD8055 inhibits the clonogenic function of human cord blood-derived HPCs. Human cord blood lineage negative (Lin- CB) cells were isolated using a human HSPC enrichment kit (BD Biosciences) by depleting cells expressing myeloid-, erythroid-, and T- and B-lineage makers according to the manufacturer's instructions. CFU assays were performed to assess the number and functions of human HPCs. (A) The effects of AZD on CFU-GM colony production are shown. (B) The effects of AZD on BFU-E colony production are shown. (C) The effects of AZD on CFU-GEMM colony production are shown.∗p < 0.05; ∗∗p < 0.01; ∗∗∗p < 0.001 vs. DMSO vehicle control. [file 561404.f1.pdf]
